# Molecular Mechanism of VSV-Vectored ASFV Vaccine Activating Immune Response in DCs

**DOI:** 10.3390/vetsci12010036

**Published:** 2025-01-09

**Authors:** Yunyun Ma, Junjun Shao, Wei Liu, Shandian Gao, Guangqing Zhou, Xuefeng Qi, Huiyun Chang

**Affiliations:** 1 State Key Laboratory for Animal Disease Control and Prevention, Lanzhou Veterinary Research Institute, Chinese Academy of Agricultural Sciences, Lanzhou 730030, China; 82101219310@caas.cn (Y.M.); shaojunjun@caas.cn (J.S.); liuwei10@caas.cn (W.L.); gaoshandian@caas.cn (S.G.); zhouguangqing@caas.cn (G.Z.); 2 College of Veterinary Medicine, Northwest A&F University, Yangling 712100, China

**Keywords:** dendritic cells, antigen presentation, recombinant viruses, immune response, ASFV

## Abstract

The vesicular stomatitis virus (VSV)-vectored African swine fever virus (ASFV) vaccine can induce efficient immune response, but the potential mechanism remains unsolved. In order to investigate the efficacy of recombinant viruses (VSV-p35, VSV-p72)-mediated dendritic cells (DCs) maturation and the mechanism of inducing T-cell immune response, the functional effects of recombinant viruses on DC activation and target antigens presentation were explored in this study. The results showed that surface-marked molecules (CD80, CD86, CD40, and MHC-II) and secreted cytokines (IL-4, TNF-α, IFN-γ) were highly expressed in the recombinant virus-infected DCs. In addition, the co-culture results of recombinant virus-treated DCs with naive T cells showed that the Th1- and Th17-type responses were effectively activated. Taken together, the study indicated that the VSV-vectored ASFV vaccine activated the maturation of DCs and the Th1- and Th17-type immune response, which provided a theoretical basis for the development of novel ASF vaccines.

## Simple Summary

African swine fever (ASF), a highly contagious disease caused by the ASF virus (ASFV), causes severe economic losses around domestic pigs and wild boars. In this study, we found that VSV-vectored ASFV vaccine effectively promoted the activation and maturation of BMDCs. The matured BMDCs secreted various inflammatory and chemotactic factors to stimulate the proliferation and activation of T cells and induce Th1- and Th17-type immune responses. Our research revealed the immune response mechanism of VSV-vectored ASFV vaccine induction in BMDCs, which provided more of a theoretical basis for VSV as a vaccine vector.

## 1. Introduction

African swine fever (ASF) is a highly lethal and contagious disease in pigs caused by the African swine fever virus (ASFV), which primarily infects domestic pigs and wild boars. It has a mortality rate of up to 100%, thereby resulting in huge economic losses in the pig farming industry since ASF outbreaks occurred in Asian and European countries in 2018–2019 [1]. Currently, vaccination is considered the most effective measure for preventing and controlling ASF. However, the lack of clinically available commercial vaccines is mainly attributed to the insufficient understanding of virus evasion of host innate and adaptive immune mechanisms as well as the function of viral proteins that can trigger protective immune responses [2]. Therefore, it is urgent to conduct in-depth research on the immune protection mechanism and pathogenesis of African swine fever virus (ASFV) infection, thereby providing novel insights and a theoretical foundation for the rational design of safe and efficient ASF vaccines.

Since the outbreak of ASF, significant progress has been made in ASFV vaccines development. A previous study showed that it did not provide strong immunological protection for weaner pigs, while the standard ASF inactivated vaccination produced certain antibodies [3]. After immunization, animals can receive immunological protection against homologous strains via attenuated live vaccines or gene-deficient vaccines, but these treatments have serious side effects and increase the likelihood of viral virulence recurrence [4,5,6]. In the meantime, despite being supposed to offer great safety, the subunit vaccines’ immunological effectiveness was inconsistent [5,7,8]. In contrast, live-vector vaccines possessed safety and efficacy and simultaneously stimulated the production of strong humoral immunity, cellular immunity, and even mucosal immunity [9,10,11,12]. So, the live-vector vaccines have become an ideal option for the development of ASFV vaccines. Currently, virus-based vector vaccines have been widely reported, and they utilize different replication mechanisms to induce cell death as well as facilitating antigen uptake and cross-presentation by dendritic cells (DCs). Furthermore, they also possess the potential to directly infect antigen-presenting cells (APCs) and elicit strong and effective immune responses. Vesicular stomatitis virus (VSV) is a promising oncolytic virus and vaccine vector that can synthesize specific antigens through a reverse genetic system and maintain high levels for a long time in cells [13]. The VSV M protein either exerted an antiviral response by inhibiting host gene expression or promoted virus replication via effectively inhibiting the synthesis of type I interferon and other antiviral gene production [14,15]. M protein mutations attenuated the ability to suppress the innate immune response but had little effect on virus assembly and replication, thereby reducing the pathogenicity of VSV in vivo [16,17,18]. The chimera of p30 and p54, known as VSV-p35 and VSV-p72, respectively, were constructed in our previous work by using two VSV recombinant variants with M gene mutations that carried the encoding ASFV p72 or p35 genes [19]. However, the mechanisms of VSV-vectored ASFV vaccine to induce immune responses in mice remain still unclear.

In the in-depth exploration of ASFV vaccine development, it has been found that the protective effect of T-cell-mediated immune response is crucial to control ASFV infection even without specific antibodies against ASFV [20,21,22,23,24,25,26]. DCs, as the most effective APCs for initiating T cells, played a crucial role in triggering T-cell responses and controlling viral infection, but their function in generating effective immunity is still unclear after being vaccinated with viral vector vaccines. Prior research revealed that domestic pigs exhibited robust humoral and cellular immune responses to poxvirus and adenovirus vector vaccines expressing ASFV p30, p54, p72, and pp62 or A151R, E119L, B602L, EP402R, B438L, K205R, and A104R genes [27,28]. Furthermore, pigs developed particular antibodies and T-cell responses in response to the ASFV component antigens transported by MVA [28]. According to Goatley et al., domestic pigs inoculated with eight ASFV gene pools have 100% resistance against virulent ASFV challenge [29]. Although many viral vectors have been extensively evaluated as preclinical candidate vaccines, the activation mechanism of the protective immune responses is yet unknown. DCs recognized pathogen-associated molecular patterns (PAMPs) through surface pattern recognition receptors (PRRs) and further triggered PRRs to induce DC maturation. Finally, antigen-specific initial T cells were activated and polarized adaptive immune responses to activate effector cells that are suitable for eliminating pathogens [30,31]. Given the results about the crucial role of DCs in activating antiviral responses, here we investigated the functional effects of VSV-p35 and VSV-p72 on DC activation and the mechanism of inducing T-cell immune response. The results demonstrated that VSV-p35 or VSV-p72 activated DC maturation and Th1/17 immune responses, which provides more of a theoretical basis for VSV as an ideal ASF vaccine vector.

## 2. Materials and Methods

### 2.1. Animals, Viruses, and Cell Lines

The 8-week-old specific pathogen-free female C57BL/6 mice were purchased from the Experimental Animal Center of Lanzhou Veterinary Research Institute (LVRI), Chinese Academy of Agricultural Sciences. The recombinant viruses, VSV-p35 and VSV-p72, were constructed and rescued as previously described [19]. VSV-rwt, a vector virus, was stored in our laboratory.

Bone marrow-derived dendritic cells (BMDCs) from female C57BL/6 mice were prepared according to the published protocol [32]. The spleen lymphocytes from the C57BL/6 mice vaccinated with the recombinant viruses VSV-p35 or VSV-p72, or with a mixture of VSV-p35 and VSV-p72 (named VSV-p35+p72), were isolated using Ficoll plus 1.077 (Solarbio, Beijing, China), and then CD4+T cells were isolated using mouse CD4+T cell isolation kit (Solarbio, Beijing, China) according to manufacturer’s instruction. The yielded cells were cultured in RPMI 1640 medium (Gibco, Carlsbad, CA, USA) supplemented with 10% fetal bovine sera (FBS, Thermo Fisher Scientific, Waltham, MA, USA), 100 U/mL penicillin (Thermo Fisher Scientific), and 100 μg/mL streptomycin solution (Thermo Fisher Scientific) at 37 °C with 5% CO_2_.

### 2.2. The Preparation of BMDCs

The BMDCs were isolated from the female C57BL/6 mice. In brief, the mice were euthanized, then the thigh bones were obtained in a sterile manner and rinsed with PBS, and then, supernatants with bone marrow cells were filtered with 70 µm cell strainers. The cells pellet was re-suspended with 4 mL red blood cell lysis solution (Solarbio, Beijing, China) at room temperature (RT) for 3 min after being centrifuged at 500× *g* for 5 min. An amount of 6 mL sterile PBS buffer was added to stop the lysis, and then the supernatants were removed by centrifugation at 500× *g* for 5 min. The cells were re-suspended with RPMI 1640 medium (Gibco, Carlsbad, CA, USA) with 10% FBS and 100 U/mL penicillin (Thermo Fisher Scientific, Waltham, MA, USA) and 100 μg/mL streptomycin solution (Thermo Fisher Scientific, Waltham, MA, USA), and then, cells were seeded into a 12-well plate at a density of 1 × 10^6^ cells per well. Subsequently, a final concentration of 20 ng/mL rmGM-CSF and 10 ng/mL rmIL-4 (Abcam, Cambridge, UK) was added. Half of the culture medium was replaced with the fresh RPMI 1640 culture medium at intervals of 2 days. On day 7 post-culture, the cells were harvested for flow cytometry and ELISA analysis.

### 2.3. Purification and Identification of BMDCs

The cultured BMDCs were centrifuged at 500× *g* for 5 min and re-suspended in 100 µL PBS. Subsequently, the cell density was adjusted to 1 × 10^6^ and 2 µL Phycoerythrin (PE) labeled CD11c were added and incubated for 30 min at 4 °C in the dark. Meanwhile, mouse IgG1 к isotype mAb was used as a control. The CD11c positively stained cells were sorted using flow cytometry (Beckman Coulter, Brea, CA, USA) and analyzed for cells by FlowJo software version 10.6.1 (BD Biosciences, Franklin Lakes, NJ, USA).

### 2.4. Real-Time Quantitative PCR (RT-qPCR)

To assess the infected capability of recombinant viruses in BMDCs and the impact on BMDCs’ function, the collected BMDCs were transferred to 12-well plates at a density of 1 × 10^6^ cells per well. Then, the cells were infected with 0.1 MOI or 1 MOI of VSV-p35, VSV-p72, or VSV-p35+p72 for 12 h, 24 h, and 48 h, respectively. Total RNA was extracted by using Trizol reagent (Thermo Fisher Scientific, Waltham, MA, USA). VSV-rwt, hiVSV-rwt (heat inactivated VSV-rwt at 65 °C for 30 min) and PBS were set up as control groups. The gene expression was detected using RT-qPCR, and the primers were designed and synthesized (Table 1).

The VSV N gene was amplified with PrimeScript™ One Step RT-PCR Kit (Takara, Dalian, China) and cloned into the pMD19-T vector to construct a standard plasmid. The standard curve was generated by 10-fold dilutions of plasmid and by performing absolute quantitative detection of VSV genome copies using One Step TB Green PrimeScript^TM^ RT-PCR Kit.

As directed by the manufacturer, RNA was quantified using RT-qPCR with the One Step TB Green PrimeScript^TM^ RT-qPCR Kit II (Takara, Dalian, China). The following is the reaction program: 42 °C for 5 min and 95 °C for 10 s, followed by 40 cycles of 95 °C for 5 s and 60 °C for 30 s. The β-actin was set to the reference gene, and the relative mRNA expression for each gene was calculated using the 2^−△△CT^ method. 

### 2.5. Western Blotting (WB)

Ice-cold RIPA lysis buffer (Solarbio, Beijing, China) was used to extract the total proteins from infected BMDCs and subjected to conduct WB analysis. In brief, cultured BMDCs were infected with 0.1 MOI of VSV-p35, VSV-p72, or VSV-p35+p72 for 24 h, respectively. Cell lysates were centrifuged at 13,000× *g* for 10 min. After collecting the supernatants, then combined with 4 × loading buffer and boiled for 15 min. Following analysis on a 10% SDS-PAGE polyacrylamide gel, the cellular proteins were deposited onto PVDF membranes. After blocking the membranes for 2 h at RT using PBST supplemented with 5% skim milk, the membranes were incubated for a whole night at 4 °C with rabbit anti-VSV-G tag antibody (diluted at a ratio of 1:1000). The membranes were then treated for 1 h at RT with goat anti-rabbit antibody that had been diluted at a ratio of 1:5000 and labeled with horse radish peroxidase (HRP). A chemiluminescence reagent was then used to view the membranes, and a FluorChem E system (ECL, Thermo Fisher Scientific, Waltham, MA, USA) was used to analyze the results.

### 2.6. Flow Cytometry Assay

In order to evaluate the maturation and differentiation of BMDCs after being infected with recombinant viruses, the molecular surface markers of BMDCs were detected by monoclonal antibodies, including PE-CD11c, FITC-CD86, APC-CD80, APC-CD40, and FITC-MHC-II mAb (BD Biosciences, San Diego, CA, USA). In brief, the cells were stained with 2 µL each mAb mentioned above for 30 min at 4 °C in the dark. Subsequently, the stained cell populations were analyzed by flow cytometry (Beckman Coulter, Brea, CA, USA).

To further assess whether recombinant virus infection can cause apoptosis of BMDCs, apoptosis assay was carried out according to previously mentioned instructions [33]. Briefly, the infected BMDCs were collected and washed with PBS by centrifugation at 500× *g* for 5 min. The cells were re-suspended with PBS and stained with 2 µL PE-CD11c for 30 min at 4 °C in the dark. Subsequently, the cells were stained with 1 μL of Fixable Viability Dye eFluor™ 780 (Thermo Fisher Scientific, 65-0865-14) for 30 min at 4 °C in the dark, and then washed with flow cytometry staining buffer. Next, 5 µL of APC-Annexin V (BD Biosciences, 550474) were added into 100 µL cell suspension to incubate for 15 min on ice in the dark. Finally, flow cytometry analysis was conducted after washing cells with 1 × combining buffer solution in the dark.

In addition, the ability of matured BMDCs to activate T cells was evaluated, including the phenotypes of lymphocytes and the expression levels of cytokines. Briefly, the infected BMDCs were co-cultured with spleen lymphocytes (DC:T = 1:10) for 12 h, 24 h, and 48 h, respectively. The cells were collected and washed with PBS by centrifugation at 500× *g* for 5 min, and then were incubated with 2 µL FITC-CD3, APC-CD4, and PE-CD8 for 30 min at 4 °C in the dark. The permeabilization buffer was added and incubated for additional 30 min at 4 °C in the dark. Next, the cells were washed with 1 mL of BD perm/wash^TM^ buffer for 5 min in the dark. Finally, the cells were re-suspended in 100 µL PBS and incubated with 2 µL PE-IL-4, PE-IL-17A, PE-IFN-γ, PE-TNF-α mAb (BD Biosciences, San Diego, CA, USA) for 45 min at 4 °C in the dark, respectively. The cell populations were analyzed by flow cytometry.

### 2.7. Detection of Cytokine and Lymphocyte Proliferation Level

The level of cytokines in cell supernatant was detected after the cells were infected with recombinant viruses for 12 h, 24 h, and 48 h at 37 °C. Cytokine measurements of IL-12p70, IFN-γ, IL-4, IL-10, IL-8, and TNF-α were performed using commercial ELISA kit according to the manufacturer’s instructions (Beijing Solarbio Science & Technology Co., Ltd., Beijing, China). Briefly, the standard curve was prepared by serially diluting the standard. Standards and the cell supernatants were added to antibody-coated 96-well microplates, while the reagent diluent was used as a blank control. Subsequently, 50 µL of 100-fold diluted biotin conjugate was added, followed by streptavidin-HRP. Finally, TMB substrate was added, and the reaction was halted with stop solution. The value of optical density was determined at 450 nm. The corresponding concentration was calculated using constructed standard curve.

The lymphocyte proliferation was assessed by Cell Counting Kit-8 kit (CCK-8, Dojindo, Japan) according to the manufacturer’s instructions. The BMDCs were infected with recombinant viruses for 24 h, then mixed with spleen lymphocytes (DC:T = 1:10) and seeded in triplicate into 96-well plates at density of 1 × 10^6^ to incubate at 37 °C with 5% CO_2_ for 12 h, 24 h, and 48 h, respectively. The positive, negative, and blank control groups were Concanavalin A (Sigma-Aldrich, MO, USA), uninfected cells, and RPMI 1640 medium, in turn. Each infection group was stimulated per well with 0.1 MOI of the corresponding recombinant viruses. Each well received the CCK-8 reagent, which was then incubated for 4 h at 37 °C. Finally, the absorbance of OD_450_ was measured and calculated using the following formula: the stimulation index SI = (treatment group OD_450_ − blank control OD_450_)/(negative control OD_450_ − blank control OD_450_).

### 2.8. Evaluation of BMDCs’ Antigen Uptake Ability

FITC-Dextran at 1 mg/mL was added to the recombinant viruses-infected BMDCs, and then cells were incubated at 37 °C or 4 °C for 2 h. Next, the cells were re-suspended with PBS. The mean fluorescence intensity (MFI) was analyzed by FlowJo software version 10.6.1 (BD Biosciences, USA). The calculation formula is as follows: ΔMFI = MFI (37 °C treatment group) − MFI (4 °C negative group).

### 2.9. Detection of BMDCs’ Migration Ability

The BMDCs were treated with 0.1 MOI of VSV-p35, VSV-p72, or VSV-p35+p72 and counted after being washed and re-suspended with PBS. RPMI 1640 culture medium containing different concentrations of CCL19 and CCL21 (concentrations of 10 ng/mL, 50 ng/mL, and 100 ng/mL, respectively) were added to the lower layer of transwell chamber (Corning, NY, USA). The 8.0 μm upper chamber was inserted into the lower chamber and incubated for 4 h at 37 °C. We removed the upper chamber and collected the lower chamber cells (migrating BMDCs) for counting. Migration rate = Lower chamber cells/whole cells × 100%.

### 2.10. Cells Viability Assay

The BMDCs were infected with 0.1 MOI of VSV-p35, VSV-p72, or VSV-p35+p72 and adjusted at density of 1 × 10^5^ cells per well into a 96-well plate at 37 °C for 12 h, 24 h, and 48 h, respectively. The PBS group was set as negative control. The CCK-8 reagent was added and incubated for 4 h at 37 °C. Finally, the value of OD_450_ was measured using a microplate reader (Thermo Fisher Scientific, Waltham, MA, USA). The cells’ viability was calculated according to the following formula: cell viability (%) = treatment group OD_450_/negative control OD_450_.

### 2.11. Indirect Immunofluorescence Assay (IFA)

The infected BMDCs were incubated for 24 h at 37 °C to perform immunofluorescence detection. The cells were first fixed for 30 min at RT using 4% paraformaldehyde solution, and then were rinsed five times with PBS. Cells were then permeabilized with 0.25% Triton-X100 for 20 min, blocked with 5% BSA at RT for 1 h, then incubated with ASFV p72 or p30 monoclonal antibody (diluted at a ratio of 1:1000) for an entire night at 4 °C. The cells were then treated for 1 h at 37 °C in the dark with the goat anti-mouse antibody tagged with fluorescence (diluted at a ratio of 1:5000). The cells were then examined under a fluorescence microscope (Leica, Wetzlar, Germany) after being treated with DAPI for 10 min.

### 2.12. Statistical Analysis

Every experiment was conducted three times, and the mean ± standard error (SEM) was used to depict the results in the figures. GraphPad Prism 7.0 software was used to determine the statistical associations between the experimental and control groups using a two-way ANOVA with Bonferroni’s multiple comparison test. A *p*-value of less than 0.05 was deemed statistically significant.

## 3. Results

### 3.1. The Functional Effects of Recombinant Viruses on BMDCs

#### 3.1.1. Phenotypic Alterations of BMDCs Following Infection with Recombinant Viruses

The proportion of cultured cells expressing CD11c reached 79.39% by flow cytometry analysis on day 7, fulfilling the purity requirements of the subsequent experiments, as shown in Figure S1. RT-qPCR and WB results confirmed that the recombinant viruses can infect and replicate effectively in BMDCs as shown in Figure S2A–C.

**Figure 1:**
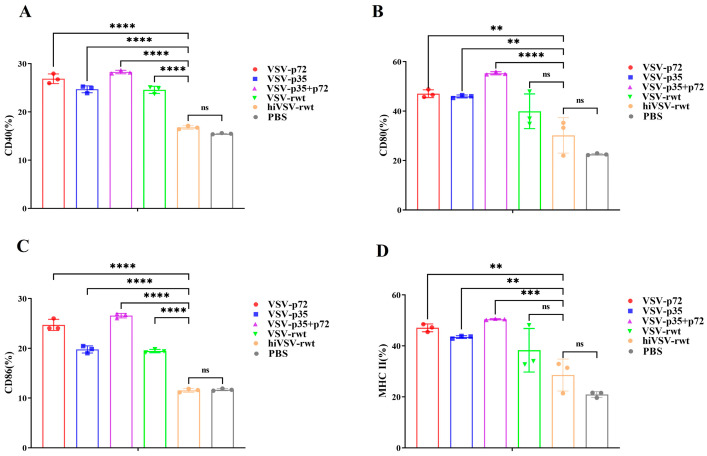
Phenotypic alterations in infected BMDCs. (**A**–**D**) Bar graph shows the percentages of the surface maturation markers CD40, CD80, CD86, and MHC-II in BMDCs from the different groups after being treated with recombinant viruses (MOI = 0.1) at 24 h, respectively. The results are shown as the mean ± SEM from three replicates per group. ** *p* < 0.01, *** *p* < 0.001, **** *p* < 0.0001, ns: not significant.

To explore whether recombinant viruses can induce BMDC maturation, the BMDCs were infected with 0.1 MOI of VSV-p72, VSV-p35, or VSV-p35+p72 for 24 h. The flow cytometry analysis revealed that the expression of CD40, CD80, CD86, and MHC-II in the recombinant virus-infected group was significantly up-regulated compared with that in the hiVSV-rwt and PBS control groups (*p* < 0.001, Figure S3 and Figure 1). The expression levels of CD80 and MHC-II were significantly higher than those of CD40 and CD86 in infected BMDCs (*p* < 0.001), which indicated a pronounced phenotypic maturation of the BMDCs. Although the expression levels of CD80 and MHC-II in the hiVSV-rwt group were higher than in the PBS group, there was no statistical difference (*p* > 0.05). These results indicated that the recombinant viruses significantly promoted BMDCs’ maturation.

#### 3.1.2. Detection of Cytokines of BMDCs After Recombinant Virus Infection

**Figure 2:**
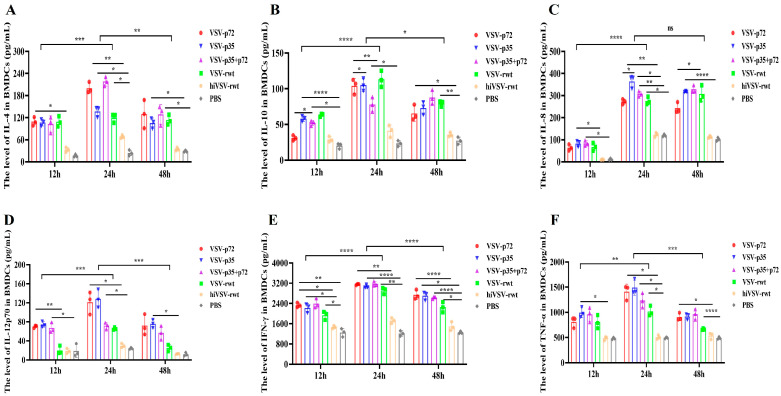
The cytokine secretion in infected BMDCs. (**A**–**F**) The secretion levels of IL-4, IL-10, IL-8, IL-12p70, IFN-γ, and TNF-α in BMDCs treated with recombinant viruses (MOI = 0.1) for 12 h, 24 h, and 48 h, respectively. The results are shown as the mean ± SEM from three replicates per group. * *p* < 0.05, ** *p* < 0.01, *** *p* < 0.001, **** *p* < 0.0001, ns: not significant.

Some cytokines and chemokines played important roles in the process of BMDC maturation and activation. Therefore, the cytokine levels of the BMDCs infected with recombinant viruses were detected with the commercial ELISA kits. As shown in Figure 2, the ELISA results showed that the levels of IL-4, IL-10, IL-8, IFN-γ, IL-12p70, and TNF-α in the infected groups were significantly higher than that those in the hiVSV-rwt and PBS control groups (*p* < 0.05). Intriguingly, IFN-γ and TNF-α levels were higher in the infected BMDCs compared with the level of IL-4, IL-10, IL-8, and IL-12p70. In addition, the secretion level of cytokines in the BMDCs was significantly increased initially, then showed a gradually decreased tendency from 12 h to 48 h, and peaked at 24 h. These above results indicated that the recombinant viruses significantly promoted the secretion of cytokines in BMDCs.

#### 3.1.3. TLRs Expression of BMDCs Infected with Recombinant Viruses

**Figure 3:**
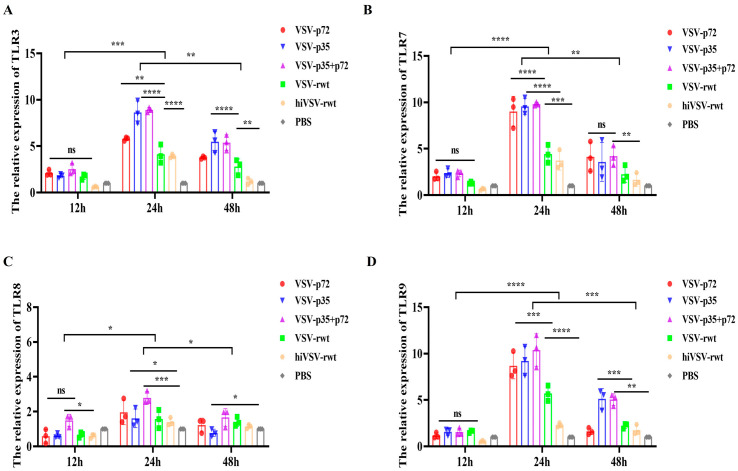
TLR expression in BMDCs infected with recombinant viruses. (**A**–**D**) The mRNA expression levels of TLR3, TLR7, TLR8, and TLR9 in BMDCs infected with recombinant viruses (MOI = 0.1) for 12 h, 24 h, and 48 h, respectively. The results are shown as the mean ± SEM from three replicates per group. * *p* < 0.05, ** *p* < 0.01, *** *p* < 0.001, **** *p* < 0.0001, ns: not significant.

Toll-like receptors (TLRs) recognize PAMPs to initiate APCs maturation as a crucial step and promote the proliferation and polarization of T cells. As shown in Figure 3, the expression levels of TLR3, TLR7, TLR8, and TLR9 increased initially and then gradually decreased from 12 to 48 h, peaking at 24 h in the infected groups, with significant differences compared with the hiVSV-rwt and PBS control groups (*p* < 0.001). Furthermore, the expression levels of TLR3, TLR7, TLR8, and TLR9 in the VSV-p35+p72 group were higher than those in the VSV-p35 and VSV-p72 groups, but there was no statistical significance (*p* > 0.05). These results indicated that recombinant viruses promoted BMDCs’ maturation and activation through TLRs.

#### 3.1.4. Phagocytosis and Migration Ability Detection in BMDCs

**Figure 4:**
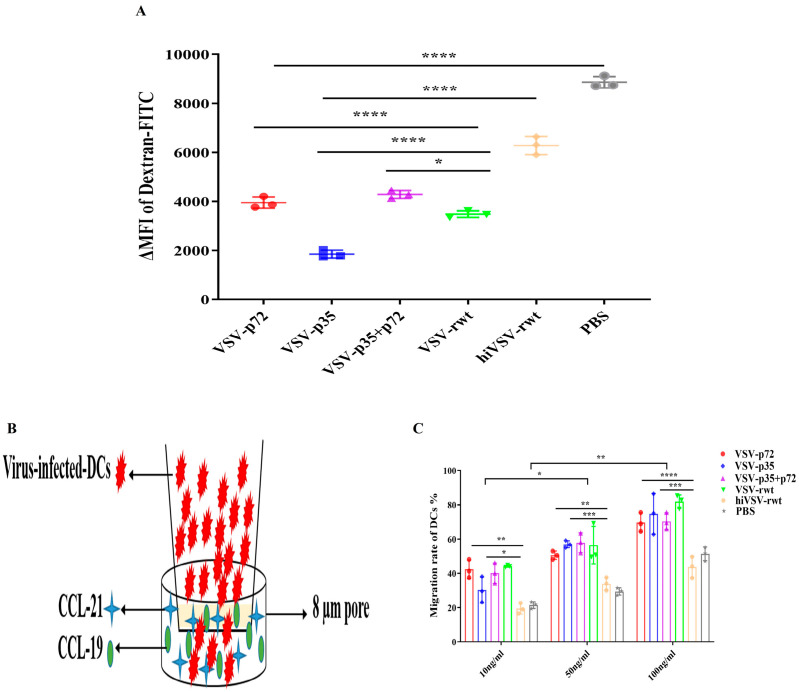
The mean fluorescence intensity of Dextran-FITC uptake by BMDCs and the migration ability of infected BMDCs. (**A**) The results are shown as the mean ± SD from three replicates per group. (**B**) The schematic diagram of the migration of infected BMDCs in transwell. (**C**) The proportion of migratory BMDCs after being stimulated with recombinant viruses (MOI = 0.1) for 24 h. The results are shown as the mean ± SEM from three replicates per group. * *p* < 0.05, ** *p* < 0.01, *** *p* < 0.001, **** *p* < 0.0001.

FITC-Dextran was used to examine the phagocytic function of BMDCs after treatment with recombinant viruses (Figure S4A,B). The phagocytic function of the infected BMDCs was significantly reduced compared with that in the hiVSV-rwt and PBS control groups (*p* < 0.001, Figure 4A). The schematic diagram of BMDCs’ migration ability is exhibited in Figure 4B. The migration ability in infected BMDCs was higher than that in the hiVSV-rwt and PBS control groups in a dose-dependent manner (*p* < 0.001, Figure 4C). These results further indicated that recombinant viruses promoted BMDCs’ maturation.

#### 3.1.5. The Apoptosis of BMDCs Infected with Recombinant Viruses

**Figure 5:**
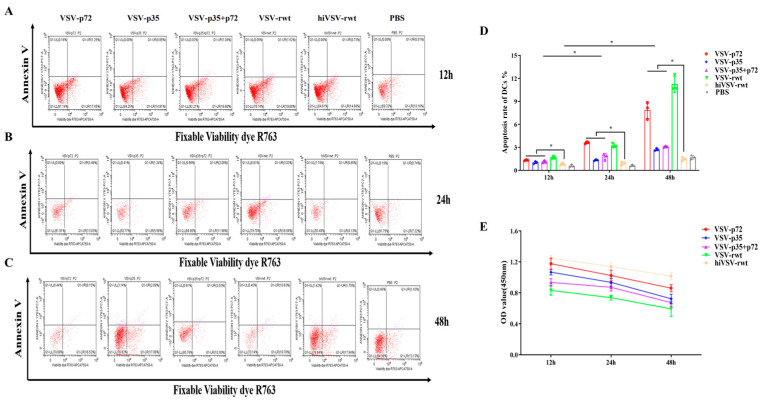
The apoptosis and cell viability of BMDCs. (**A**–**C**) The apoptosis rates of BMDCs after being stimulated with recombinant viruses (MOI = 0.1) for 12 h, 24 h, and 48 h, respectively. (**D**) Bar graph showing statistical analysis of apoptosis rates in treated BMDCs. (**E**) Cell viability of BMDCs after being stimulated with recombinant viruses (MOI = 0.1) for 12 h, 24 h, and 48 h, respectively. The results are shown as the mean ± SEM from three replicates per group. * *p* < 0.05.

To test whether recombinant viruses cause apoptosis and affect antigen presentation ability in BMDCs, the apoptosis rates in the recombinant virus-infected BMDCs were detected by flow cytometry. As shown in Figure 5A–D, the results showed that the apoptosis rate in the infected groups was gradually increased from 12 to 48 h with statistical significance compared with the hiVSV-rwt and PBS control groups (*p* < 0.05). The cell viability decreased by less than 20% at 48 h according to the CCK-8 results (Figure 5E). These results indicated that the recombinant viruses have no significant effect on the apoptosis and cell viability of BMDCs, thereby proving that the recombinant viruses did not interfere with the antigen presentation ability of the BMDCs.

#### 3.1.6. Antigen Expression of the Matured BMDCs

**Figure 6:**
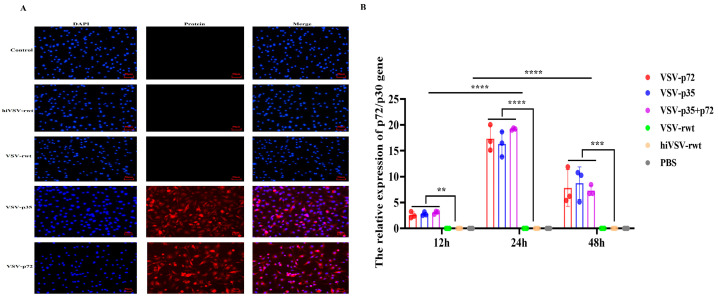
The antigen expression of the matured BMDCs. (**A**) The fluorescence expression of ASFV p72 or p30 protein in BMDCs after being stimulated with recombinant viruses (MOI = 0.1) for 24 h. (**B**) The relative mRNA expression of p72/p30 gene in BMDCs after being stimulated with recombinant viruses (MOI = 0.1) for 24 h. The results are shown as the mean ± SEM from three replicates per group. ** *p* < 0.01, *** *p* < 0.001, **** *p* < 0.0001.

To explore whether the matured BMDCs infected with recombinant viruses can effectively express target antigens, the antigen expression was detected by IFA and RT-qPCR assays. The IFA results showed that the ASFV p72 and p30 antigens were effectively expressed in the matured BMDCs (Figure 6A). RT-qPCR results showed that the expression of the ASFV p72 and p30 antigens could be detected at scheduled times. The mRNA expression of antigens in the infected groups showed a tendency of increasing initially and then decreasing; it reached a peak at 24 h and showed significant differences compared with the VSV-rwt, hiVSV-rwt, and PBS control groups (*p* < 0.001, Figure 6B). These results indicated that recombinant viruses promoted the antigen expression in the matured BMDCs.

### 3.2. The Effects of the Matured BMDCs on T Cells Activation

#### 3.2.1. T Cells Proliferation and Activation in Co-Cultured Cells

**Figure 7:**
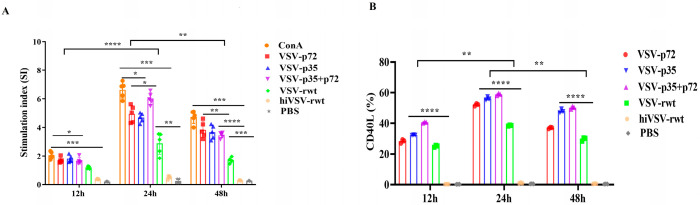
The proliferation and activation ability of T cells. (**A**) The proliferation of T cells in co-cultured cells was measured by mixed lymphocyte reaction assay when BMDCs were infected with recombinant viruses (MOI = 0.1) for 12 h, 24 h, and 48 h. (**B**) The percentage of CD40L in co-cultured cells was analyzed by flow cytometry when BMDCs were infected with recombinant viruses (MOI = 0.1) for 12 h, 24 h, and 48 h. The results are shown as the mean ± SEM from three replicates per group. * *p* < 0.05, ** *p* < 0.01, *** *p* < 0.001, **** *p* < 0.0001.

To assess the ability of recombinant viruses-primed BMDCs to stimulate T-cell proliferation and activation, the T cells’ proliferation was measured in a mixed lymphocyte culture condition. The results showed that the proliferation proportion of T cells began with an initial increase at 12 h post-co-culture, reached a peak at 24 h, and then gradually decreased. Moreover, the stimulation index of T cells in the recombinant virus infection group was significantly higher than that of both the hiVSV-rwt and PBS control groups (*p* < 0.001, Figure 7A). The robust expression of CD40L on the T cells’ surfaces further verified that the matured BMDCs triggered T-cell activation in the infected groups (Figure S5 and Figure 7B). These data provided evidence for T lymphocytes’ activation in the matured BMDCs infected with recombinant viruses.

#### 3.2.2. The Effect of the Recombinant Viruses on the Differentiation of T Lymphocyte Subsets

**Figure 8:**
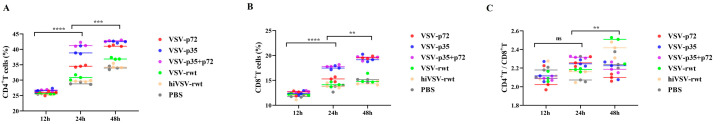
The percentage of CD3+CD4+T or CD3+CD8+T cells in co-cultured cells. (**A**,**B**) The percentage of CD4+ or CD8+ cells of CD3+T cells in co-cultured cells was measured when BMDCs were infected with recombinant viruses (MOI = 0.1) for 12 h, 24 h, and 48 h, respectively. (**C**) The ratio of CD4+T to CD8+T cells. The results are shown as the mean ± SEM from three replicates per group. ** *p* < 0.01, *** *p* < 0.001, **** *p* < 0.0001.

The proportion of lymphocyte subsets CD3+CD4+/CD3+CD8+ is an important indicator to evaluate immune response. The differentiation of T lymphocytes was detected by flow cytometry in co-cultured cells. The percentages of CD4+T cells to total CD3+T cells gradually increased from 12 to 48 h (Figure 8A and Figure S6), and the percentages of CD8+T cells to total CD3+T cells also showed a similar increasing tendency (Figure 8B and Figure S6). The percentages of CD4+T cells were significantly higher than those of CD8+ T cells (*p* < 0.01, Figure 8C), indicating that the matured BMDCs infected with recombinant viruses activated T lymphocytes.

#### 3.2.3. The Effect of the Recombinant Viruses on the Activation of CD4+T Lymphocyte Subsets

**Figure 9:**
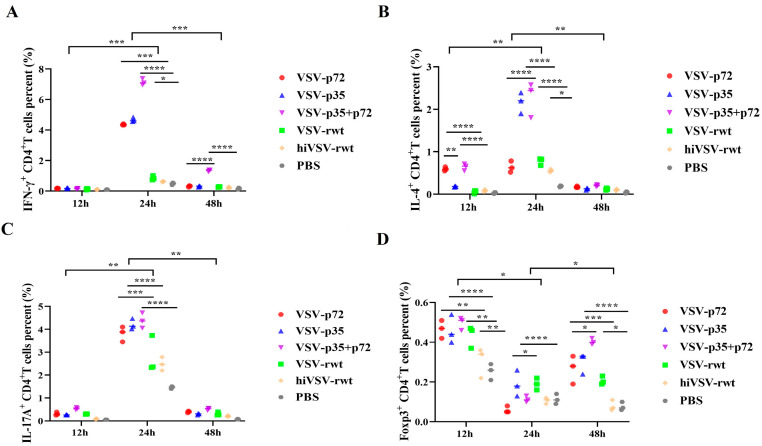
The polarization of CD4+T lymphocyte subsets. (**A**–**D**) Dot plots present individual values of the triplicates per treatment. Statistical differences among individual groups at a time and per individual group over the time points were analyzed using a two-way ANOVA with Bonferroni’s multiple comparison statistical test. * *p* < 0.05, ** *p* < 0.01, *** *p* < 0.001, **** *p* < 0.0001.

To analyze the immune type of recombinant viruses toward CD4+T cells, we detected the percentage of intracellular cytokines such as IL-4, IL-17A, IFN-γ, and Foxp3 by flow cytometry in CD4+T cells. As shown in Figure 9A–D, the percentages of IL-4+CD4+T, IL-17A+CD4+T, and IFN-γ+CD4+T were increased in the infected groups, reached a peak at 24 h, and then subsided to the base level. At 24 h, the percentage of IL-7A+CD4+T and IFN-γ+CD4+T was significantly higher than that in control groups (*p* < 0.001). Intriguingly, the percentage of Foxp3 reached the highest at 12 h and then up-regulated at 48 h again (Figure 9 and Figure S7). These results indicated that the matured BMDCs infected with recombinant viruses effectively activated T cells and maybe also stimulated the activation of Th1 and Th17 cells.

#### 3.2.4. The Expression of Transcription Factors in CD4+T Cells

We further detected the mRNA expression of transcription factors in the CD4+T cells that were co-cultured with recombinant virus-infected BMDCs for 24 h. As shown in Figure S8, there was no significant difference among all groups in terms of the mRNA expression of T-bet, GATA-3, and RORγt at 12 h (*p* > 0.05); then, they were transiently up-regulated in the infected groups and showed a significant difference compared with that in the control groups at 24 h (*p* < 0.001, Figure S8A–C). The mRNA level of Foxp3 in the infected group was significantly higher than that in the control groups at 12 h. In contrast, the mRNA level of Foxp3 subsided to the base level at 24 h in the infected groups, and then showed an increasing trend at 48 h with significant differences in the infected groups compared with the control groups (*p* < 0.001, Figure S8D). These results further confirmed that matured BMDCs are one of the major stimulators that resulted in activation of Th1- and Th17-type cells.

## 4. Discussion

The development of an available vaccine against ASFV infection to control the current pandemic is partially hampered by the poor knowledge of ASF adaptive immunity. To deeply understand the mechanism of VSV-vectored ASFV vaccines inducing adaptive immune response, we provided an intensive study of the specific immune responses in recombinant virus-infected BMDCs. We demonstrated that recombinant viruses stimulated BMDCs’ maturation to secrete cytokines (IL-4, TNF-α, IFN-γ) and induce Th1- and Th17-type immune response. These data suggested that recombinant virus-induced adaptive immunity was dominated by activating T cells, thereby stimulating the activation and proliferation of Th1 and Th17 cells against ASFV infection.

A recombinant virus carrying a genetically modified antigen has been demonstrated to be an effective approach to deliver antigens into DCs [34]. More importantly, recombinant viral vectors such as adenovirus, lentivirus, and vaccinia virus can induce DC maturation, though controversial results have also been reported [34,35,36]. VSV is a non-pathogenic negative-stranded and enveloped RNA virus that has become one of the most promising viral vectors for the development of vaccines against pathogens with high biological requirements [37,38]. VSV also triggered cytotoxic T-cell responses toward viral proteins and tumor-associated antigens as an oncolytic agent, thereby producing long-lasting anti-tumor effects [39]. The VSV M protein exhibited the function of inhibiting IFN production and shutting down protein synthesis, serving as a pivotal determinant of viral virulence. The absence of the 51st amino acid or mutations M51R, V221F, and S226R attenuated the virulence of the VSV strain [32]. A previous study confirmed that VSV_MT_ effectively induced DCs’ maturation and promoted a violent immune response by delaying cell apoptosis, secreting cytokines, and promoting the expression of the surface molecules CD80/86 and MHC-II [40]. Additionally, Boudreau et al. found the ΔM51-VSV induced DC maturation and pro-inflammatory cytokine production and particularly induced a strong type-I IFN response in DCs, which not only attenuated viral replication and spread but further improved the DCs’ function [41]. Our results regarding the induction and activation of specific immune response against ASFV by recombinant viruses also confirmed these findings. These works demonstrated that VSV, as a vaccine vector, can present exogenous antigens to BMDCs, thereby activating the BMDCs and naive T lymphocytes, generating a specific immune response to exert antiviral effect. Similarly, our results showed that the recombinant VSV with three amino acid mutations (M51R, V221F, and S226R) induced BMDC maturation and reduced the degree of BMDC apoptosis, which may cause viral virulence attenuation.

Previous research confirmed that TLR expression was induced by feline infectious peritonitis virus infection, which was consistent with our result for the expression trend of TLRs during recombinant viral infection [42]. In our study, the expression of TLR3, TLR7, and TLR9 was up-regulated at 24 h after being treated with recombinant virus but not TLR8, which indicated TLR3, TLR7, and TLR9 may participate in BMDC maturation and activation, but the potential mechanism still needs to be further explored. In the late stage of infection, the expression of TLRs gradually decreased. We inferred that this may be caused by balancing excessive immune responses, which was dominated by increasing the percentages of regulatory T cells (Tregs) and decreasing the percentages of Th1 and Th17 cells. Therefore, the result explained why the expression of Foxp3 increased in the late stage of infection in subsequent experiments. The DCs secreted crucial cytokines and chemokines when the DCs were stimulated by external factors. Particularly, the balance between TNF-α and IL-10 plays an important role in ASFV infection [43,44,45]. IFN-γ and TNF-α, as the critical cytokines, have demonstrated that can promote DCs’ activation and antigen presentation [46]. IL-8 is a functional chemotactic factor that can activate neutrophil phagocytosis and lysosomal activity and exhibits a chemotactic effect on T lymphocytes [47]. IL-12p70 has an important effect in stimulating the differentiation of immature T cells into Th1-type cells and promoting the activation of immune cells during antigen presentation, thereby enhancing cytotoxicity and activating cellular immunity [48]. The role of cytokines in the maturation of BMDCs induced by recombinant virus was further analyzed. The study confirmed that the expression of cytokines (IL-4, IL-8, IL-12p70, IFN-γ, and TNF-α) in BMDCs treated with recombination virus was significantly up-regulated and was positively correlated with the expression of MHC-II, co-stimulatory molecules CD80/86, and TLRs on the surface of BMDCs, which suggested that the secretion of TNF-α and IL-12p70 induced by the recombinant virus may be mediated by TLRs, and the potential mechanism may be related to the expression of TLR3, TLR7, and TLR9 caused by viral infection. Up-regulation of TLR3, TLR7, and TLR9 expression promoted the release of IL-4, IL-8, IL-12p70, IFN-γ, and TNF-α in BMDCs. These results indicated that the recombinant virus can recognize pathogens through TLRs to stimulate the maturation of BMDCs and increase the expression of MHC-II and co-stimulatory molecules, thus promoting the expression of related inflammatory cytokines and chemokines to protect viral invasion.

CD4+T cells can activate other types of immune cells to produce a direct immune response. When the number of CD4+T cells increases, the host can quickly activate the immune response to resist pathogens once the invasion of pathogenic microorganisms is received. In the subtypes of CD4+T cells differentiation, Th1 cells mainly secreted IFN-γ to activate effector cells and promote the clearance of pathogens. Th2 cells secreted IL-4 and IL-10 to inhibit the activation of phagocytes and the development function of Th1 cells [49]. Th17 cells mainly secreted IL-17A and IL-22 to promote neutrophil aggregation, mediate inflammatory responses, and exert regulatory effects on both immune and non-immune cells [50]. Treg cells, as a subset of CD4+T lymphocytes, play an important role in inhibiting effector T lymphocytes and IL-10/TGF-β, thereby inducing immune tolerance and regulating immune homeostasis [51,52]. Therefore, exploring the differentiation ability of recombinant viruses to induce CD4+T lymphocytes is important for explaining the protection mechanism of recombinant viruses against ASFV infection. Previous research demonstrated that the proportion of CD4+T cells in the splenic lymphocytes from mice immunized with recombinant viruses was significantly higher than that of CD8+T cells [19]. Here, we conducted an in-depth study of the immune mechanism of BMDCs treated with recombinant viruses and co-cultured with CD4+T cells. The results confirmed that the proportions of IFN-γ+CD4+T cells and IL-17A+CD4+T cells in BMDCs that were treated with recombinant virus for 24 h significantly increased, and the mRNA expression levels of T-bet and RORγt also was significantly up-regulated. But the proportion of Foxp3+CD4+T cells and the mRNA level of Foxp3 were decreased. According to the above findings, we suspected that the high expression of cytokines secreted by Th1 and Th17 cells inhibited the expression of Foxp3. Meanwhile, Th17 cells exerted immune functions by secreting IL-17A, which is a pro-inflammatory cytokine that can accelerate the inflammatory response caused by neutrophils via inducing inflammatory factors such as IFN-γ and TNF-α [53,54]. Our results found that IL-17A expression increased first and then decreased, which remained consistent with the trend of IFN-γ expression and indicated that Th1 and Th17 play an important role in resisting ASFV infection. Taken together, co-culture of matured DCs and splenic lymphocytes induced the activation, proliferation, and differentiation of T cells into Th1 and Th17 cells. Given the regulation of the splenic lymphocyte populations by activated DCs observed in this study, it may be inferred that the activation and proliferation of T cells may involve several mechanisms. These mechanisms encompass direct stimulation from activated DCs and indirect regulation by other immune cells, such as natural killer (NK) cells, macrophages, and B cells, that are activated by cytokines released by the activated DCs as well as the synergistic effects of these two types of regulation.

Our research further confirmed previous findings on the potential mechanism of recombinant viruses inducing efficient immune responses in mice, revealing that recombinant viruses can induce immune protection by stimulating relevant immune responses in the host. However, it must be pointed out that there are still some limitations in this research, especially since the results have not been further verified in the pig model. In view of the above drawbacks, the following experiments will mainly focus on evaluating the immune protection of the recombinant virus live vector vaccine prototype in pigs and further exploring the potential immune mechanism.

## 5. Conclusions

Taken together, the study found that the recombinant viruses effectively promoted the activation and maturation of BMDCs. Matured DCs stimulated the proliferation and activation of initial T cells by various mechanisms, further leading the initial CD4+T lymphocytes toward differentiation of Th1 and Th17 cells, and then inducing the host to produce a dominant Th1- and Th17-type immune response. Therefore, our research provided more of a theoretical basis for VSV as an ASF vaccine vector.

## Figures and Tables

**Table 1:** The sequences of primers used in this study.

Primers	Sequences (5′-3′)
VSV N	Forward: GGAATAAACATCGGGAAAG
	Reverse: TGGTTGCCTTTGTATCTACTT
TLR3	Forward: TCGGCAACGGTTCCTTCTCC
	Reverse: AATGCTCGCTTCAAACTCAGGTAC
TLR7	Forward: AAAGCCCTTTACCTGGATGGAAAC
	Reverse: TCGTGATGGAGAAGATGTTGTTAGC
TLR8	Forward: GGTTATGTTGGCTGCTCTGGTTC
	Reverse: TGGGATGTGGATGAAGTCCTGTA
TLR9	Forward: AACCTCAGCCACAACATTCTCAAG
	Reverse: CACCTCCAACAGTAAGTCTACGAAG
ASFV-p72	Forward: CTGCTCATGGTATCAATCTTATCGA
	Reverse: GATACCACAAGATCAGCCGT
ASFV-p30	Forward: ATCTACGCAGGACAGGGATACAC
	Reverse: GTCGTTCTTCTCGTGGATGTTCTC
T-bet	Forward: ATCACTAAGCAAGGACGGCGAATG
	Reverse: TCCACCAAGACCACATCCACAAAC
GATA-3	Forward: TCTGGAGGAGGAACGCTAATGGG
	Reverse: CGGGTCTGGATGCCTTCTTTCTTC
RORγt	Forward: TGTCCCGAGATGCTGTCAAGTTTG
	Reverse: TCCTGTTGCTGCTGCTGTTGC
Foxp3	Forward: AAGAATGCCATCCGCCACAACC
	Reverse: TACGGTCCACACTGCTCCCTTC
β-actin	Forward: CTGGCACCACACCTTCTACAATGAG
	Reverse: TGGCGTGAGGGAGAGCATAGC

## Data Availability

The data that support the findings of this study are available on request from the corresponding author.

## Supplementary Materials

The following supporting information can be downloaded at: https://www.mdpi.com/article/10.3390/vetsci12010036/s1, Figure S1: The isolation and identification of BMDCs; Figure S2: The infection capability of recombinant viruses in BMDCs; Figure S3: The expression of surface maturation markers CD40, CD80, CD86 and MHC-II in the infected BMDCs; Figure S4: The phagocytic function in BMDCs; Figure S5: The expression of CD40L in co-cultured cells was detected by flow cytometry when BMDCs were infected with recombinant viruses for 12 h, 24 h, and 48 h, respectively; Figure S6: The percentage of CD3+CD4+T or CD3+CD8+T cells in co-cultured cells was measured when BMDCs were infected with recombinant viruses for 12 h, 24 h, and 48 h, respectively; Figure S7: The activation of CD4+T lymphocyte subsets; Figure S8: The expression of transcription factors in co-cultured cells. And the Original images of Western Blotting (WB).
