# Supplementary material for: Molecular Mechanism of VSV-Vectored ASFV Vaccine Activating Immune Response in DCs
Source: Vet Sci. 2025 Jan 9;12(1):36. doi: 10.3390/vetsci12010036 (PMC11769090; doi:10.3390/vetsci12010036)
Supplement: Supplementary file 1 [file vetsci-12-00036-s001.zip › vetsci-3384777-supplementary.pdf]

Supplementary Materials

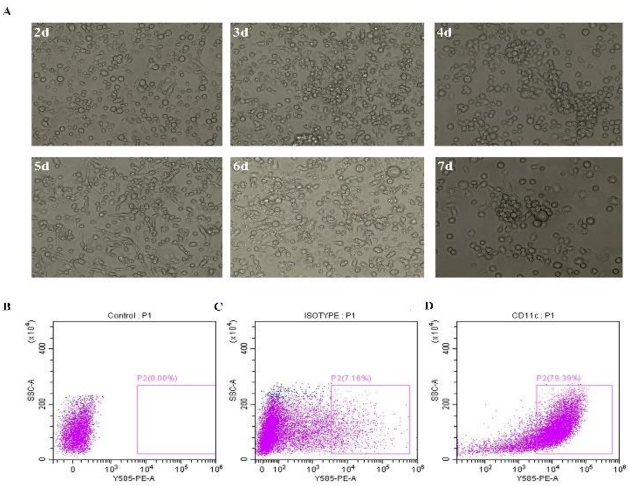

Figure S1. The isolation and identification of BMDCs. (**A**) The morphologies of BMDCs induced from mouse bone marrow-derived monocytes in *vitro* at different time points. (**B**) circle gate P2: CD11c-positive cell population of unstained BMDCs. (**C**) circle gate P2: CD11c-positive cell population of BMDCs stained with Phycoerythrin-either labeled mouse IgG1 κ isotype. (**D**) circle gate P2: CD11c-positive cell population of BMDCs stained with Phycoerythrin-either labeled CD11c.

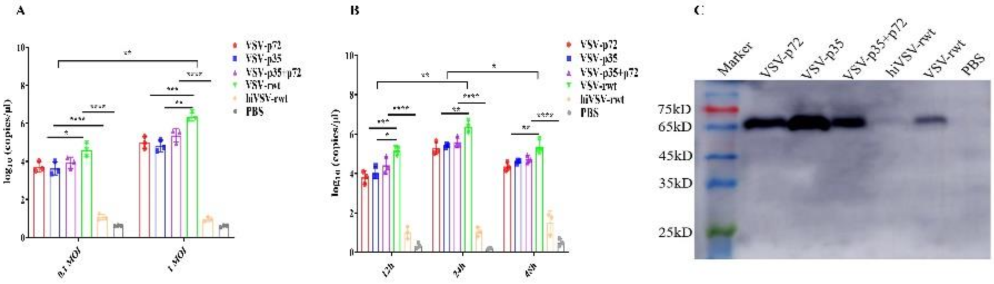

Figure S2. The infection capability of recombinant viruses in BMDCs. (**A**) The virus copies in BMDCs that infected with 0.1 MOI and 1 MOI recombinant viruses. (**B**) The virus copies in BMDCs that infected with recombinant viruses for 12 h, 24 h, and 48 h. (**C**) The expression of VSV G protein in BMDCs that infected with recombinant viruses for 24 h.

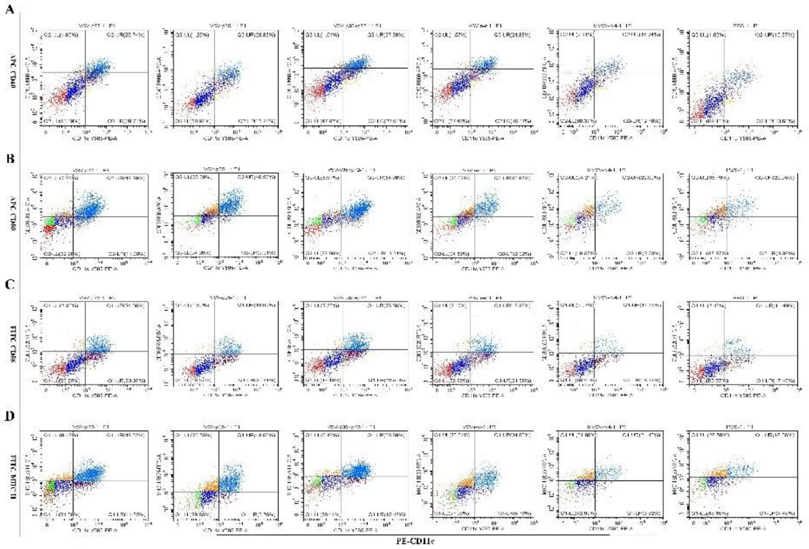

Figure S3. The expression of surface maturation markers CD40, CD80, CD86 and MHC-II in the infected BMDCs. (**A-D**) representative dot plots depicted the percentages of CD40, CD80, CD86 and MHC-II in recombinant viruses treated BMDCs for 24 h, respectively. Red represents CD11c positive cells population labeled with PE, orange represents MHC-II cells population labeled with FITC, blue represents CD86 cells population labeled with FITC, green represents CD80 cells population labeled with APC, yellow represents CD40 cells population labeled with APC.

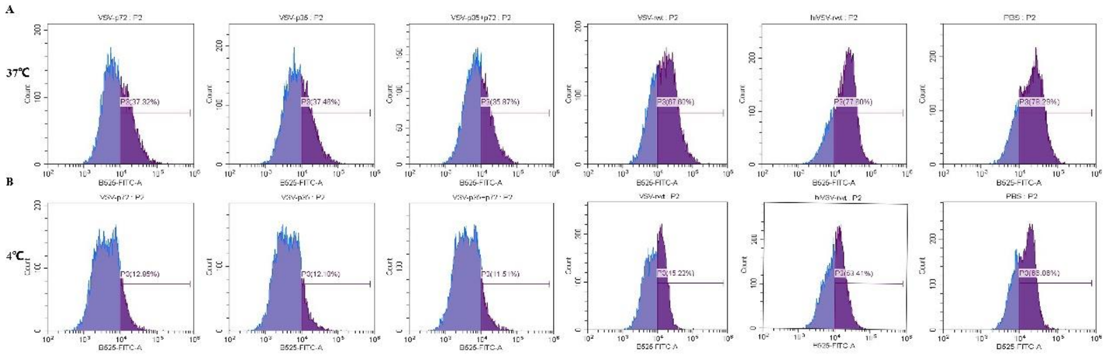

Figure S4. The phagocytic function in BMDCs. (**A, B**) the proportion of $FITC^+$DCs after being stimulated with recombinant viruses for 24 h at 37 °C or 4 °C.

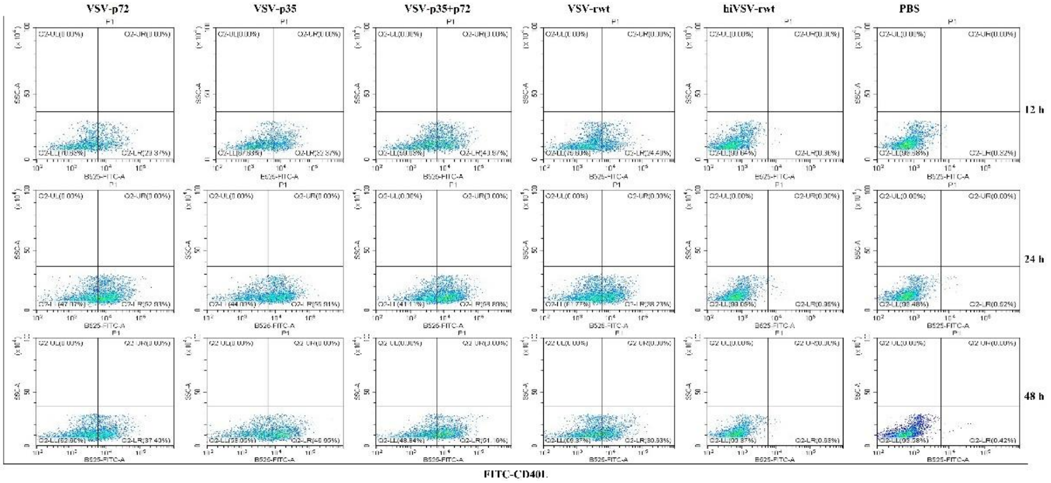

Figure S5. The expression of CD40L in co-cultured cells was detected by flow cytometry when BMDCs were infected with recombinant viruses for 12 h, 24 h, and 48 h, respectively.

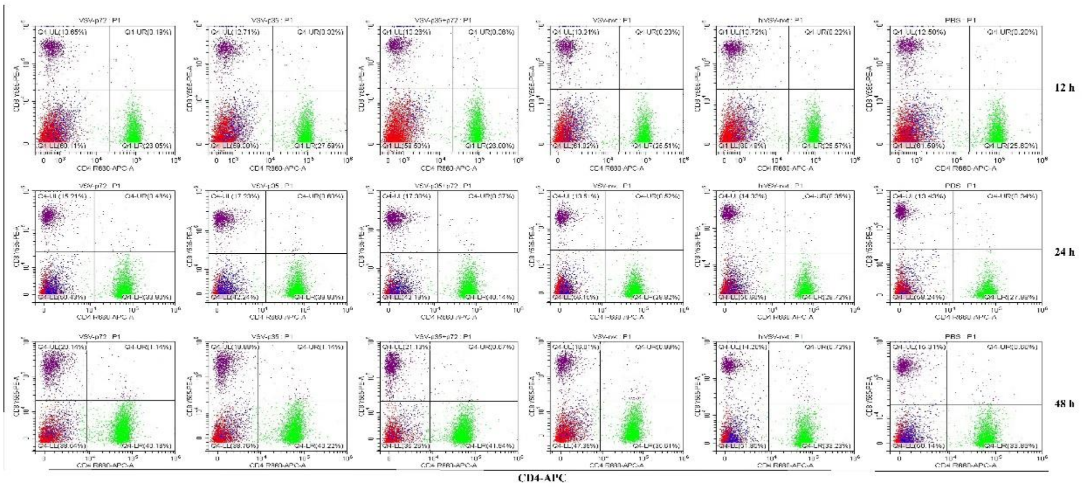

Figure S6. The percentage of $CD3^{+}CD4^{+}$T or $CD3^{+}CD8^{+}$T cells in co-cultured cells was measured when BMDCs were infected with recombinant viruses for 12 h, 24 h, and 48 h, respectively.

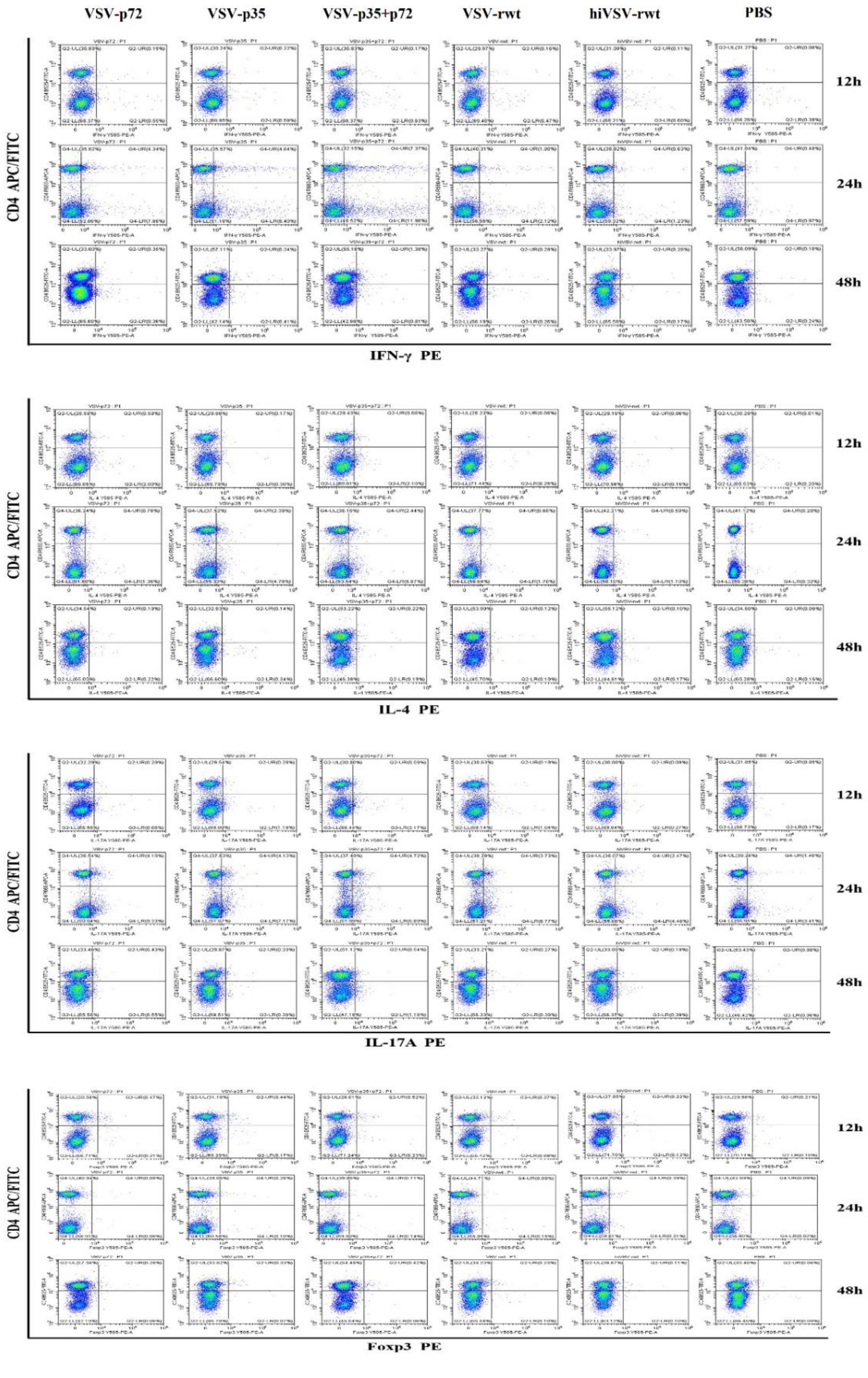

Figure S7. The activation of CD4$^+$T lymphocyte subsets. (**A-D**) The percentages of IFN-γ$^+$CD4$^+$, IL-4$^+$CD4$^+$, IL-17A$^+$CD4$^+$, and Foxp3$^+$CD4$^+$T cells in co-cultured cells were measured when BMDCs were infected with recombinant viruses at 0.1MOI for

12 h, 24 h and 48 h, respectively.

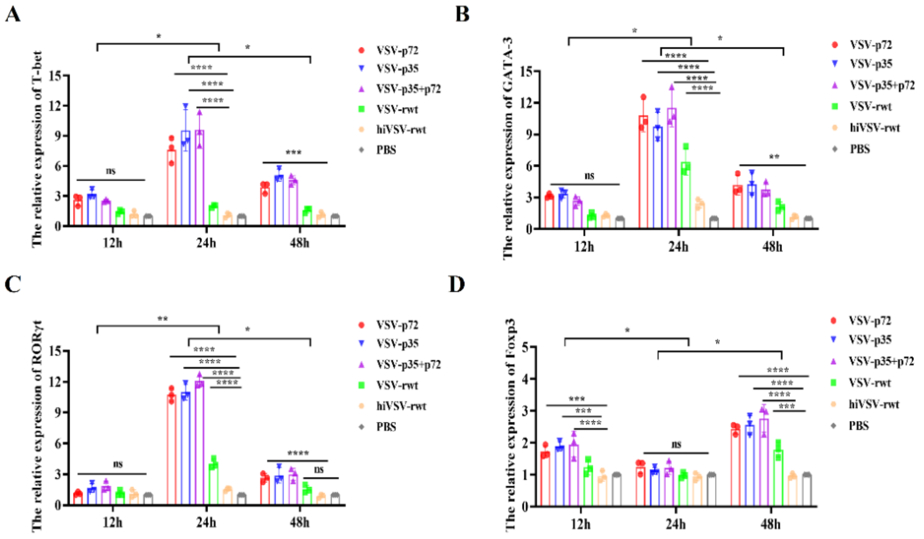

Figure S8. The expression of transcription factors in co-cultured cells. (**A-D**) The mRNA expression of T-bet, GATA-3, RORγt and Foxp3 in infected BMDCs at 12 h, 24 h and 48 h, respectively. The results showed as the mean ± SEM (n = 3). $^{*}p < 0.05$, $^{**}p < 0.01$, $^{***}p < 0.001$, $^{****}p < 0.0001$, ns: not significant.
