# Supplementary figures and images for: Molecular Mechanism of VSV-Vectored ASFV Vaccine Activating Immune Response in DCs
Source: Vet Sci. 2025 Jan 9;12(1):36. doi: 10.3390/vetsci12010036 (PMC11769090; doi:10.3390/vetsci12010036)

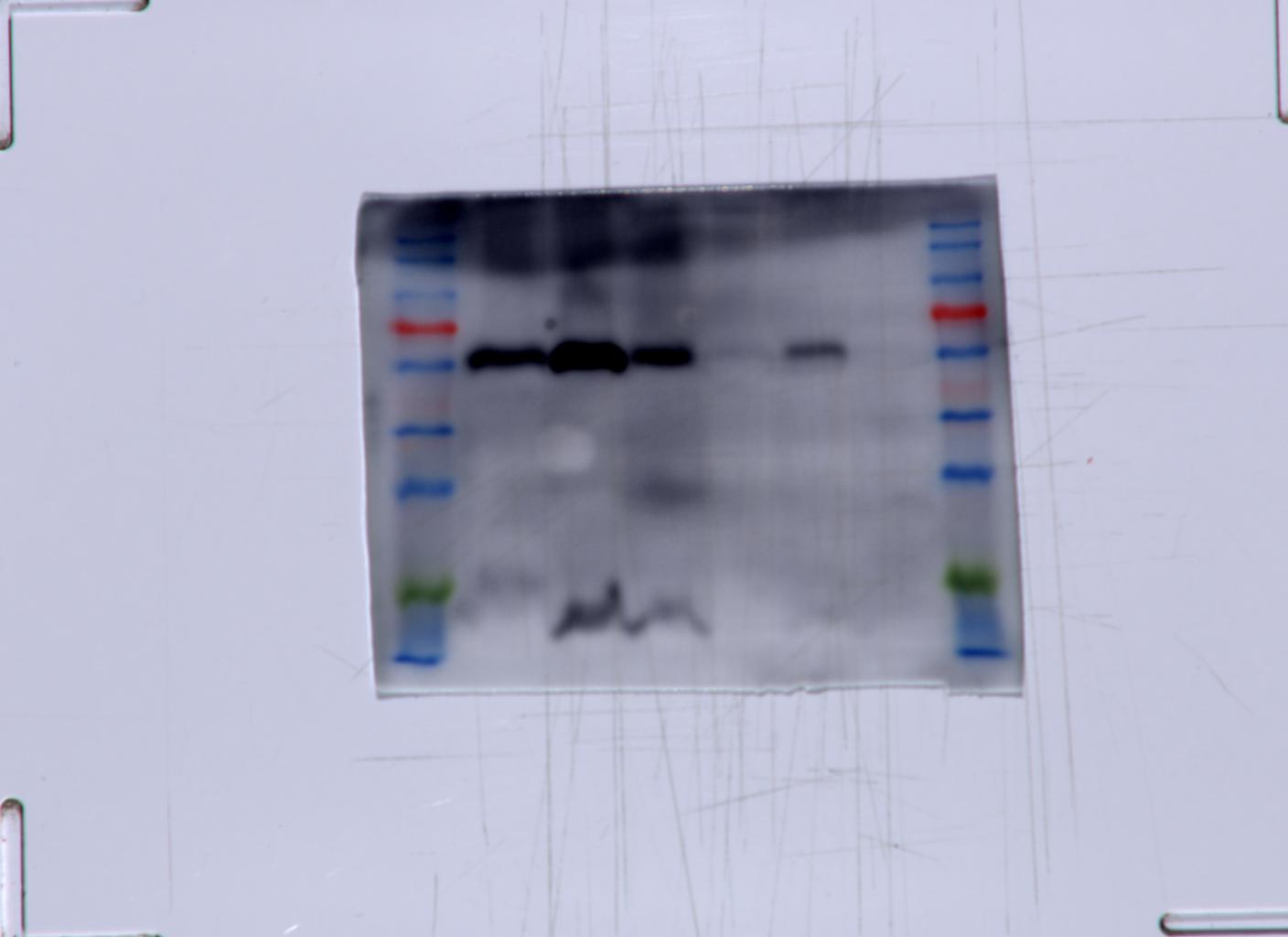

Supplement: Supplementary file 1 [file vetsci-12-00036-s001.zip › WB原始图.jpg]
